# O‐GlcNAc transferase activates stem‐like cell potential in hepatocarcinoma through O‐GlcNAcylation of eukaryotic initiation factor 4E

**DOI:** 10.1111/jcmm.14043

**Published:** 2019-01-24

**Authors:** Benjin Cao, Meng Duan, Yang Xing, Chanjuan Liu, Fan Yang, Yinan Li, Tianxiao Yang, Yuanyan Wei, Qiang Gao, Jianhai Jiang

**Affiliations:** ^1^ Key Laboratory of Glycoconjuates Research, Ministry of Public Health, Department of Biochemistry and Molecular Biology School of Basic Medical Sciences, Fudan Shanghai People’s Republic of China; ^2^ Department of Liver Surgery and Transplantation Liver Cancer Institute, Zhongshan Hospital, Fudan University Key Laboratory of Carcinogenesis and Cancer Invasion of Ministry of Education Shanghai People’s Republic of China

**Keywords:** eukaryotic initiation factor 4E, hepatocellular carcinoma, O‐GlcNAc transferase, O‐GlcNAcylation, stem‐like cell potential

## Abstract

O‐GlcNAcylation catalysed by O‐GlcNAc transferase (OGT) is a reversible post‐translational modification. O‐GlcNAcylation participates in transcription, epigenetic regulation, and intracellular signalling. Dysregulation of O‐GlcNAcylation in response to high glucose or OGT expression has been implicated in metabolic diseases and cancer. However, the underlying mechanisms by which OGT regulates hepatoma development remain largely unknown. Here, we employed the lentiviral shRNA‐based system to knockdown OGT to analyse the contribution of OGT in hepatoma cell proliferation and stem‐like cell potential. The sphere‐forming assay and western blot analysis of stem‐related gene expression were used to evaluate stem‐like cell potential of hepatoma cell. We found that the level of total O‐GlcNAcylation or OGT protein was increased in hepatocellular carcinoma. OGT activated stem‐like cell potential in hepatoma through eukaryotic initiation factor 4E (eIF4E) which bound to stem‐related gene Sox2 5'‐untranslated region. O‐GlcNAcylation of eIF4E at threonine 168 and threonine 177 protected it from degradation through proteasome pathway. Expression of eIF4E in hepatoma was determined by immunostaining in 232 HCC patients, and Kaplan‐Meier survival analysis was used to determine the correlation of eIF4E expression with prognosis. High glucose promoted stem‐like cell potential of hepatoma cell through OGT‐eIF4E axis. Collectively, our findings indicate that OGT promotes the stem‐like cell potential of hepatoma cell through O‐GlcNAcylation of eIF4E. These results provide a mechanism of HCC development and a cue between the pathogenesis of HCC and high glucose condition.

## 1 INTRODUCTION

Hepatocellular carcinoma (HCC) is the most common primary liver malignancy worldwide, especially in developing countries.^1^, ^2^, ^3^ Formation of HCC results from multiple risk factors, including HBV or HCV infection, cirrhosis, excessive alcohol consumption, and a variety of genetic factors.^4^ Increasing evidences reveal that diabetes is associated with an increased HCC incidence and has been far more than HBV, HCV, or alcoholic liver disease. A study in Taiwan populations has reported that the incidence of HCC was twice higher in the diabetes patients group compared with the non‐diabetes patients.^5^ Similarly, HBEl‐Serag research showed that the proportion of HCC patients with diabetes (43%) was significantly greater than non‐cancer controls (19%).^6^ However, the potential mechanism for the pathogenesis of HCC with diabetes is incompletely understood.

High glucose increases intracellular concentrations of UDP‐GlcNAc, resulting in increased global O‐GlcNAcylation.^7^ O‐GlcNAc is an O‐linked‐β‐N‐acetylglucosamine moiety attached to the residue of serine or threonine on nuclear or cytoplasmic proteins.^8^ The addition of O‐GlcNAc to proteins is catalysed by O‐GlcNAc transferase (OGT), and its removal is catalysed by O‐GlcNAcase (OGA).^9^ O‐GlcNAcylation regulates such diverse cellular processes as nutrient sensing, cell cycle progression, transcription, translation, epigenetic regulations, and protein‐protein interactions.^10^, ^11^, ^12^, ^13^ O‐GlcNAcylation is believed to play a role in a variety of signalling cascades that mediate glucose homoeostasis and stress responses.^14^

Elevated O‐GlcNAcylation has been described in different cancer types including breast, prostate, liver, colon, lung, and chronic lymphocytic leukaemia.^15^, ^16^, ^17^, ^18^, ^19^ These studies indicate that high O‐GlcNAcylation seems to be a general feature of cancer cells and contributes to tumorigenesis. In hepatoma, Zhu et  al reported that O‐GlcNAcylation was higher in cancer tissues compared to normal tissues. Moreover, O‐GlcNAcylation was higher again in patients diagnosed a recurrence of hepatocarcinoma.^17^ Considering that high glucose increases global O‐GlcNAcylation, elucidating the role of O‐GlcNAcylation in hepatoma progression helps to improving our understanding of the association between diabetes and HCC. However, the contribution of O‐GlcNAcylation in hepatoma development remains largely unknown.

In this study, we investigated the roles of OGT in hepatoma development. We found that OGT was highly expressed in hepatocellular carcinoma. OGT activated stem‐like cell potential of hepatoma cell through O‐GlcNAcylation of eukaryotic initiation factor 4E. Eukaryotic initiation factor 4E, a key translation factor, bound to stem‐related gene Sox2 5'‐untranslated region. High glucose promoted stem‐like cell potential of hepatoma cell through OGT‐eIF4E axis. Our findings indicate that OGT promotes the stem‐like cell potential of hepatoma cell through O‐GlcNAcylation of eIF4E, providing a mechanism of HCC development.

## 2 MATERIALS AND METHODS

### 2.1 Human tumour tissues

Hepatocarcinoma tissues and matching tumour adjacent normal tissues were obtained from the ZhongShan Hospital, Shanghai. The procedures related to human subjects were approved by the Ethics Committee of the Institutes of Biomedical Sciences, Fudan University. Experiments were undertaken with the understanding and written consent of each subject.

### 2.2 Plasmids and transfection

PCRs were performed to amplify eIF4E cDNA and inserted eIF4E cDNA into Lv‐Flag vector with Flag tag at N‐terminal region, HA tag at C‐terminal region. eIF4E single mutant (T168A or T177A) and double mutant (T168, 177A) were constructed. The pCMV‐OGT‐myc plasmid was provided as described previously.^20^ The pLKO.1‐OGT shRNA plasmids were constructed. Transient transfection was performed following standard protocols of Lipofectamine 2000 (Invitrogen, 11668). The sequence of oligonucleotide primers of wild type and mutants of eIF4E construction were presented, as follows: WT eIF4E‐forward primer: 5ʹ‐CGGGATCCATGGATTACAAGGATGACGACGATAAGATGGCGACTGTCGAACCG‐3ʹ, WT eIF4E‐reverse primer: 5ʹ‐ ACGCGTCGACTTAAGCGTAATCTGGAACATCGTATGGGTAAACAACAAACCTATTTTTAG‐3ʹ; eIF4E(T168A)‐forward primer: 5ʹ‐GCTGAATGTGAAAACAGAGAAGCTGTTACA‐3ʹ, eIF4E(T168A)‐reverse primer: 5ʹ‐GTTTTCACATTCAGCAGTCCATATTGCTAT‐3ʹ; eIF4E(T177A)‐forward primer: 5ʹ‐ GCACATATAGGGAGGGTATACAAGGAAAGG‐3ʹ, eIF4E(T177A)‐reverse primer: 5ʹ‐ CCTCCCTATATGTGCAACAGCTTCTCTGTT‐3ʹ. The oligonucleotide primers for OGT shRNA sequences were presented, as follows: control shRNA‐forward primer: 5ʹ‐ CCGGTCCTAAGGTTAAGTCGCCCTCGCTCGAGCGAGGGCGACTTAACCTTAGGTTTTTG‐3ʹ, control shRNA‐reverse primer: 5ʹ‐ AATTCAAAAACCTAAGGTTAAGTCGCCCTCGCTCGAGCGAGGGCGACTTAACCTTAGGA‐3ʹ; OGT shRNA1‐forward primer: 5ʹ‐ CCGGTGGATGCTTATATCAATTTAGGCTCGAGCCTAAATTGATATAAGCATCCTTTTTG‐3ʹ, OGT shRNA1‐reverse primer: 5ʹ‐ AATTCAAAAAGGATGCTTATATCAATTTAGGCTCGAGCCTAAATTGATATAAGCATCCA‐3ʹ; OGT shRNA2‐forward primer: 5ʹ‐ CCGGTGCACAATCCTGATAAATTTGACTCGAGTCAAATTTATCAGGATTGTGCTTTTTG‐3ʹ, OGT shRNA2‐ reverse primer: 5ʹ‐ AATTCAAAAAGCACAATCCTGATAAATTTGACTCGAGTCAAATTTATCAGGATTGTGCA‐3ʹ.

### 2.3 Western blotting

Cell lysates were produced by 1x sample loading buffer, boiled for 10 minutes and separated by SDS‐PAGE. Then, the gels were transferred to PVDF (Roche), blocked 2 hours in PBST (PBS with 0.1% v/v Tween‐20) containing 5% w/v BSA (Amresco) and incubated overnight in PBST/5% w/v BSA with primary antibody. Then, blots were incubated 90 minutes with horseradish peroxidase‐conjugated mouse or rabbit secondary antibodies (1:3000, Santa Cruz Biotechnology respectively) in PBST/5% w/v BSA and visualized by chemiluminescence. To probe for O‐GlcNAc, blots were blocked 3 hours in 5% w/v milk in TBST (Tris‐Buffered Saline, 0.1% v/v Tween‐20), incubated overnight at 4°C in TBST/5% w/v BSA with O‐GlcNAc‐specific mouse antibody (RL2), then for 1 hour with secondary anti‐mouse IgG horseradish peroxidase conjugated antibody in TBST/5% w/v milk, and visualized by chemiluminescence. The primary antibodies used were listed, as follows: RL2 (Thermo, MA1‐072), eIF4E (BD, 610269), eIF4E (CST, 2067), OGT (CST, 5368), HA (CST, 3724), eIF4A (CST, 2013), eIF4G (CST, 2469), myc (CST, 2278), KLF4 (CST, 4038), Sox2 (R&D, AF2018), OCT4 (R&D, AF1759), ubiquitin (Santa Cruz, sc‐8017), and β‐actin (Sigma, A1978). Dilution ratio was performed according to the antibody instruction. CST, Cell Signaling Technology; BD, BD Biosciences; Santa Cruz, Santa Cruz Biotechnology; Thermo, Themo Fisher Scientific; R&D, R&D Systems; Sigma, Sigma‐Aldrich.

### 2.4 Mapping of O‐GlcNAc site using mass spectrometry

To map O‐GlcNAc sites, Nano‐LC‐ESI‐MS/MS was performed as previously described.^21^ Overexpressed wild‐type Flag‐eIF4E proteins in HEK293T cells were purified using ANTI‐FLAG M2 affinity gel (Sigma‐Aldrich, A2220) and subjected to SDS‐PAGE.

### 2.5 RNA‐ChIP assays

RNA‐ChIP assays were performed using RNA ChIP‐IT Kit (Activemotif, 53024) according to manufacturer's instructions. In brief, Huh7 cells were collected and lysed. The supernatants was incubated with eIF4E antibody (BD Biosciences, 610269) and rocked at 4°C overnight. Total RNAs were eluted and carried out with RT‐PCR. The oligonucleotide primers for RNA‐ChIP assays were presented, as follows: Sox2‐forward primer: 5ʹ‐ AAAGTATCAGGAGTTGTCAAGGCAGAG‐3ʹ, Sox2‐reverse primer: 5ʹ‐ GAGGCAAACTGGAATCAGGATCAAA‐3ʹ; KLF4‐forward primer: 5ʹ‐ GGACCTACTTACTCGCCTTGCTGATTG‐3ʹ, KLF4‐reverse primer: 5ʹ‐ TGGCCGAGATCCTTCTTCTTTGGA‐3ʹ; OCT4‐forward primer: 5ʹ‐ TCCAGTCCCAGGACATCAAAGCT‐3ʹ, OCT4‐reverse primer: 5ʹ‐ GCAGATGGTCGTTTGGCTGAATA‐3ʹ.

### 2.6 Tissue microarray and immunohistochemistry

Tissue microarray was constructed and immunohistochemistry was carried out as described previously.^22^, ^23^ The eIF4E and O‐GlcNAcylation immunostaining intensities were semi‐quantitatively scored as: 0, negative; 1, weak; 2, moderate; and 3, strong. All samples were anonymized and independently scored by two investigators. In case of disagreement, the slides were re‐examined and a consensus was reached by the observers.

### 2.7 sWGA‐affinity purification

Cells and tissues were lysed with RIPA lysis buffer (150 mM NaCl, 50 mM Tris (pH = 7.4), 1 mM EDTA, 1% Nonidet P‐40) and lysates were incubated with agarose‐conjugated sWGA (Vector Laboratories, AL‐1023S) for overnight at 4°C. For control of specificity, 50 mM GlcNAc (Sigma‐Aldrich, A3286) was added. Precipitates were washed three times with lysis buffer and proteins were eluted by boiling in 1x SDS sample buffer.

### 2.8 Immunoprecipitation assays

Cells were washed with cold phosphate‐buffered saline (PBS), then lysed on ice with RIPA lysis buffer (10 mM Tris/HCl (pH 7.4), 150 mM NaCl, 1% Triton X‐100 (v/v), 0.5% sodium deoxycholate (w/v), 0.1% sodium dodecyl sulfate (w/v), and protease inhibitors) for immunoprecipitation. The cell extracts were then centrifuged at 12 000 *g* for 10 minutes at 4°C. The supernatants were pre‐cleared with sepharose‐labelled protein G (Roche) for 2 hours. The beads were discarded after a 1 minute centrifugation at 2500 *g*, and the supernatants was incubated with interest primary antibodies and rocked at 4°C overnight. Controls for immunoprecipitation specificities were performed with normal mouse or rabbit IgG (Santa Cruz Biotechnology).

### 2.9 Cell proliferation assays

Cell proliferation assays was analysed using the commercial Cell Counting Kit (CCK8) in accordance with the manufacturer's instructions. In brief, cells were seeded onto 96‐well plates (Corning) at a density of 5 × 10^3^ cells/well, and incubated for 2 hours to allow cell adherence to the plate. CCK8 reagents (Dojindo, CK04) were added to each well and incubated for 2 hours at 37°C. Absorbance at 450 nm was measured using Microplate Reader (Bio‐Tech Instruments, USA). The results were plotted as means ± SD of three separate experiments.

### 2.10 Tumorsphere assays

For spheroid assays, cells were digested to single‐cell and seeded (200 cells/well) in conditional culture (Dulbecco's modified Eagle's and F12 media supplemented with 50x B27, 2 μg/mL heparin, 20 ng/mL EGF and 20 ng/mL FGF‐2) in 96‐well Ultra‐Low Attachment Microplates (Corning, 3474). The number of spheroids was measured and analysed 12 days after seeding.

### 2.11 Flow cytometry

1.5 × 10^6^ cells were collected and washed with cold PBS twice times by centrifugation at 300 *g* for 10 minutes at 4°C. The phycoerythrin (PE)‐conjugated CD133/1 clone AC133 antibody and mouse IgG isotype control antibody (Miltenyi Biotec) were incubated with cells for 10 minutes on ice under dark according to the manufacturer's protocol. Samples were analysed by using a FACS apparatus MoFlo XDP (Beckman Coulter, US).

### 2.12 Statistical analyses

Statistical analysis of the data was calculated by using two‐tailed Student's *t* tests (*P < 0.05, **P < 0.01) on GraphPad Prism. All values included in figures represent mean ± SD Error bars represent SD. Data are representative of at least three independent experiments.

## 3 RESULTS

### 3.1 Elevated level of O‐GlcNAcylation and OGT in HCC

**Figure 1:**
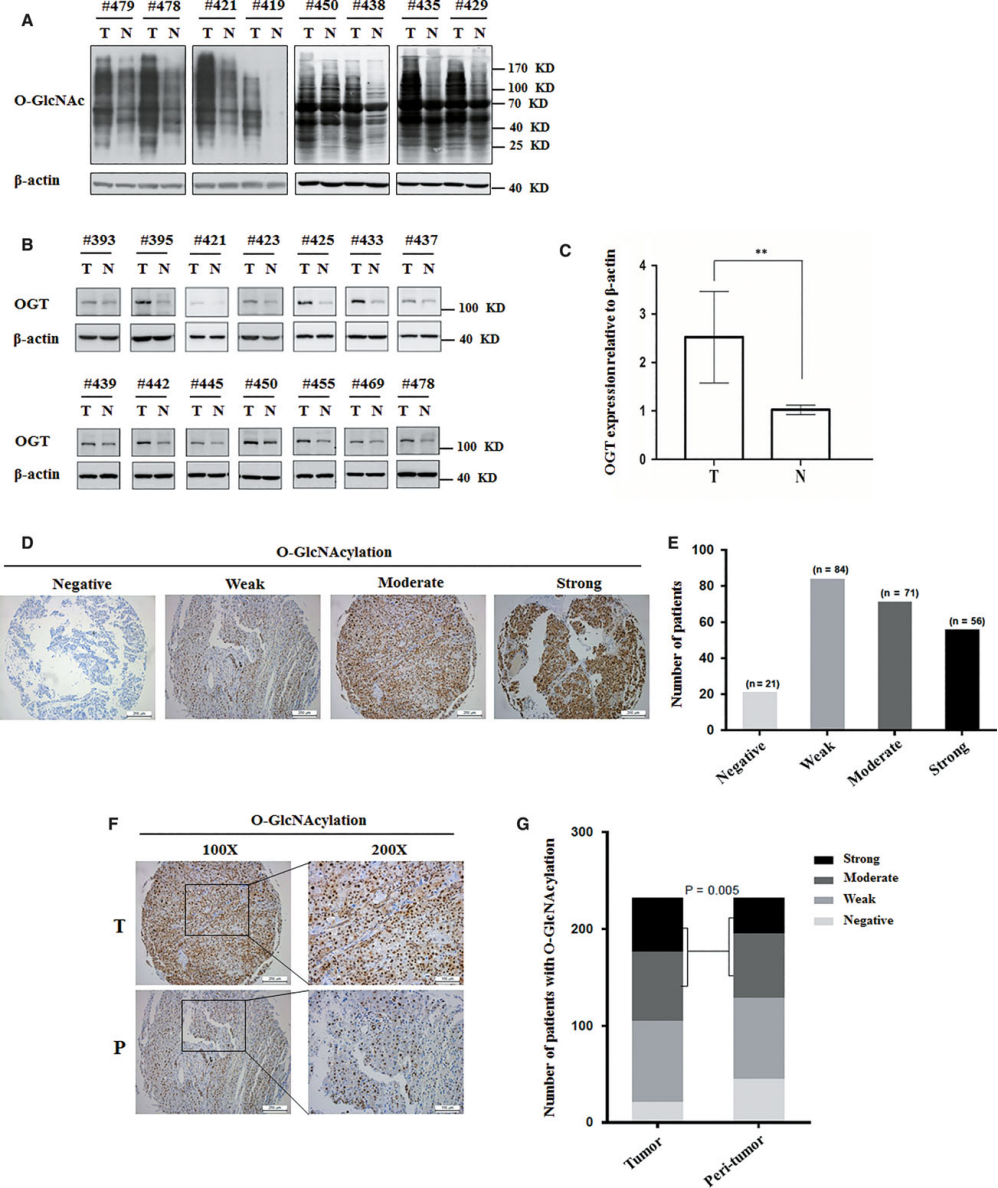
The difference of O‐GlcNAcylation and OGT protein level between hepatoma tissues and adjacent normal liver tissues. A, The level of total O‐GlcNAcylation was analysed in eight paired HCC specimens with their corresponding non‐cancerous specimens by western blotting. β‐actin expression was served as a loading control. B, OGT protein expression levels were analysed in 14 paired HCC specimens by western blotting. β‐actin expression was served as a loading control. C, OGT values were calculated from images in B. Data represent mean *± *SD of at least three independent experiments. **P < 0.01. D, Representative immunohistochemical staining of O‐GlcNAcylation in liver cancer tissues. E, Number of different staining of O‐GlcNAcylation patients was analysed. F, Representative immunohistochemical staining of O‐GlcNAcylation in liver cancer tissues and peri‐tumour liver tissues. G, Quantitative analysis of the level of O‐GlcNAcylation in liver cancer tissue microarrays showed that the expression of O‐GlcNAcylation was increased in liver cancer (*P = *0.005). Immunohistochemical staining was estimated, as follows: negative: staining intensity ≤10%; weak: staining intensity in 10%‐20%; moderate: staining intensity in 20%‐50%; strong: staining intensity > 50%. T: tumour sample; N: non‐cancerous sample

To learn the pathophysiologic significance of O‐GlcNAcylation in hepatoma development, we first determined the level of O‐GlcNAcylation and OGT in human hepatocellular carcinoma and adjacent normal tissues. The level of O‐GlcNAcylation was significantly increased in hepatoma tissues compared to paired adjacent liver tissues (Figure 1A). Accordingly, OGT expression was significantly increased in hepatoma tissues compared to paired adjacent liver tissues (Figure 1B‐C). We further investigated this phenomenon by immunohistochemistry analysis in 232 paired HCC tissues. The majority of hepatoma tissues had been high expression of O‐GlcNAcylation (Figure 1D‐E). The intensity of O‐GlcNAcylation immunostaining was markedly enhanced in hepatoma tissues compared to that in the peri‐tumour tissues (Figure 1F‐G). Thus, these results indicate that the level of O‐GlcNAcylation and OGT protein are increased in hepatoma.

### 3.2 Knockdown of OGT suppresses cell growth and stem‐like cell potential of hepatoma cell

**Figure 2:**
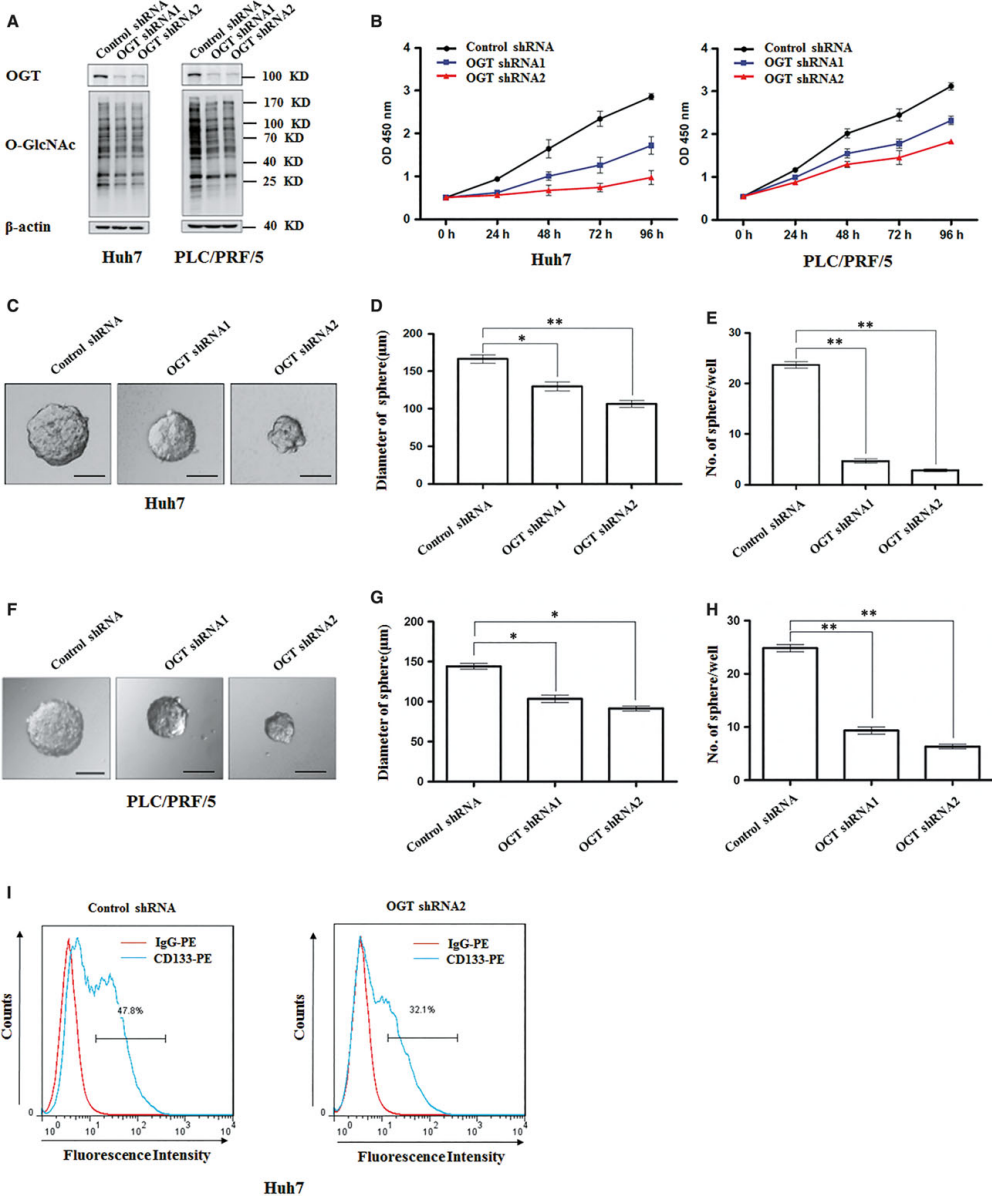
Knockdown of OGT inhibits the proliferation and tumorsphere formation of hepatoma cell in vitro. A, Huh7 and PLC/PRF/5 cells were infected with control shRNA, OGT shRNA1, and OGT shRNA2 lentivirus. Protein lysates were collected after 48 h for immunoblot analysis with indicated antibodies. β‐actin expression was served as a loading control. B, Cell proliferation of Huh7 and PLC/PRF/5 cells infected with control shRNA, OGT shRNA1, and OGT shRNA2 lentivirus were measured with CCK8 assay. (C‐H) Huh7 and PLC/PRF/5 cells, respectively, infected with the indicated lentivirus were seeded into 96‐well plates. After 12 d, tumorsphere were counted and quantified. Representative images of sphere (scale bars, 100 μm) were shown (C, F). The diameter of sphere (D, G) and number of sphere (E, H) were count. Data represent mean *± *SD of at least three independent experiments. *P < 0.05; **P < 0.01. I, Huh7 cells expressing either control shRNA or OGT shRNA2 were incubated with PE‐labelled anti‐AC133 antibody. The percentages of CD133^+^ cells in graphs were analysed by flow cytometry. Red line, control IgG staining; blue line, CD133 staining. Representative flow cytometry data from three independent experiments were shown

The high expression of O‐GlcNAcylation in hepatoma promoted us to examine the contribution of O‐GlcNAcylation in HCC development. We used a lentiviral shRNA‐based system to evaluate the requirement for O‐GlcNAcylation in hepatoma development. Western blot showed that the level of OGT and total O‐GlcNAcylation were obviously decreased by OGT shRNA lentivirus in Huh7 and PLC/PRF/5 cells (Figure 2A). We first examined the effect of OGT knockdown on hepatoma cell growth. CCK8 assay showed that OGT knockdown reduced cell proliferation in Huh7 and PLC/PRF/5 cells (Figure 2B). Considering that cancer stem cell is responsible for the initiation of HCC.^24^ We next examined the contribution of OGT in stem‐like cell potential in hepatocarcinoma. The sphere‐forming assays have been widely used to evaluate the self‐renewal ability of cancer stem cell.^25^ Knockdown of OGT obviously decreased the diameter and number of tumorsphere in Huh7 and PLC/PRF/5 cells in conditional culture (Figure 2C‐H). We further demonstrate that down‐regulation of OGT expression decreased stem‐like cell potential in Huh7 cells. The population of cancer stem cells was examined by flow cytometry using stem cell marker, like CD133. Flow cytometry analysis showed that the percentage of CD133^+^ cells decreased from 47.8% to 32.1% after OGT knockdown in Huh7 cells (Figure 2I). Meanwhile, some reports have showed that stem‐like potential proteins, such as Sox2, OCT4, and KLF4, which can augment stem‐cell function. And these proteins can be used as stemness‐related markers.^26^, ^27^, ^28^, ^29^ The expression of stem‐like cell potential proteins (Sox2, OCT4 and KLF4) were also reduced in Huh7 cells with OGT knockdown (Figure 6J). Together, these data suggest that OGT promotes cell proliferation and activates stem‐like cell potential in hepatocarcinoma.

### 3.3 eIF4E is O‐GlcNAcylated in hepatoma

**Figure 3:**
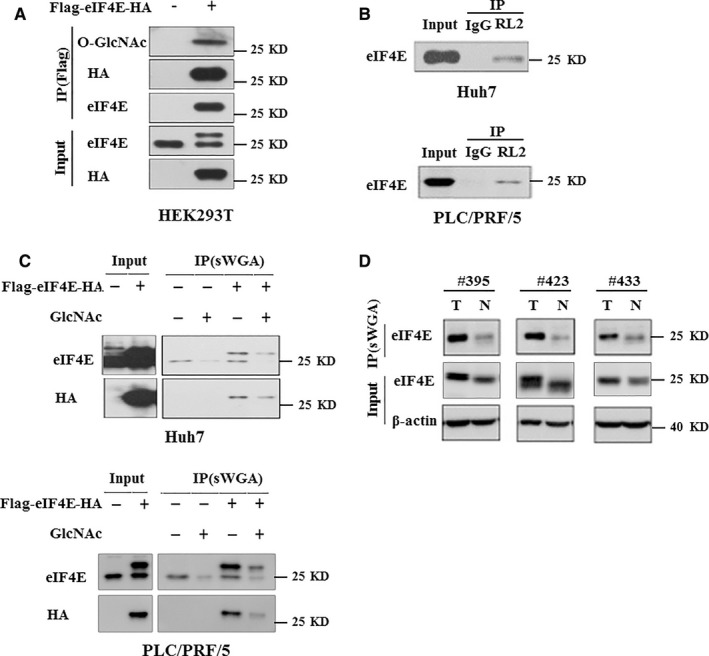
eIF4E is modified with O‐GlcNAc in vivo and in vitro. A, Exogenous Flag‐eIF4E‐HA was O‐GlcNAcylated in HEK293T cells. B, Endogenous eIF4E was O‐GlcNAcylated in Huh7 and PLC/PRF/5 cells. O‐GlcNAc modified protein obtained from cell extracts were analysed by immunoblotting for eIF4E. C, Exogenous eIF4E was O‐GlcNAcylated in Huh7 and PLC/PRF/5 cells using another method. Total O‐GlcNAc‐modified proteins were precipitated with succinylated wheat germ agglutinin (sWGA), a lectin binding specifically to O‐GlcNAc. The precipitates were then analysed by western blotting for eIF4E. For control, monosaccharide inhibitor GlcNAc (*N*‐Acetyl‐D‐glucosamine, 50 mM) was added during sWGA lectin‐affinity purification. The specificity of the sWGA lectin is illustrated by effectively competing the lectin with GlcNAc. D, Paired HCC specimens lysates were subjected to sWGA‐affinity purification and the precipitates were analysed by western blotting for eIF4E. β‐actin expression was served as a loading control

We next aimed to explore the mechanisms underlying OGT promotes stem‐like cell potential in hepatocarcinoma. Previous study reported that O‐GlcNAc modification of ribosomal subunits contributed to the translational machinery.^12^ Of numerous eukaryotic initiation factors in translational machinery, eIF4E is a key player in the regulation of translation initiation and is required for the recruitment of specific mRNAs to the ribosome.^30^ eIF4E has been reported to regulate the self‐renewal of glioma‐initiating cell.^31^ These findings promoted us to investigate whether eIF4E was O‐GlcNAcylated. First, we examined whether exogenous eIF4E could be O‐GlcNAcylated. Flag‐tagged eIF4E purified from HEK293T cells was examined by immunoblotting using the anti‐O‐GlcNAc antibody (RL2). Western blot assay showed that exogenous eIF4E could be O‐GlcNAcylated (Figure 3A). Next, we examined whether O‐GlcNAcylation of eIF4E also occurs in hepatoma cell. Total O‐GlcNAc modified protein were immunoprecipitated from cell extracts of Huh7 and PLC/PRF/5 cells using RL2 antibody. Endogenous eIF4E was successfully detected in precipitates (Figure 3B). Accordingly, exogenous eIF4E has been O‐GlcNAc modified in Huh7 and PLC/PRF/5 cells using succinylated wheat germ agglutinin (sWGA) for affinity purification (Figure 3C). To further determine whether eIF4E was O‐GlcNAcylated in the liver cancer samples, we used sWGA for affinity purification of hepatocellular carcinoma and adjacent normal tissues lysates. The precipitates were successfully probed with an antibody against eIF4E (Figure 3D). Thus, eIF4E is O‐GlcNAcylated in hepatoma.

### 3.4 Mutation of eIF4E O‐GlcNAc sites at threonine 168 and threonine 177 reduces its protein stability

**Figure 4:**
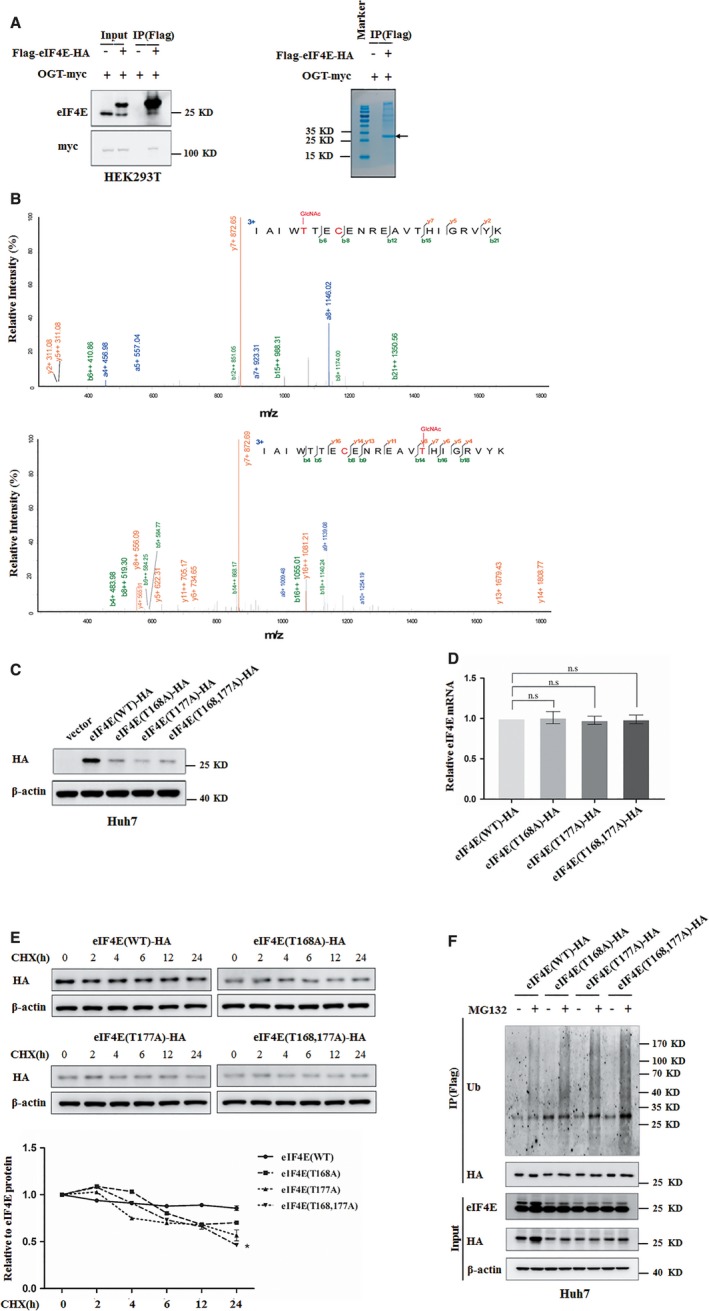
eIF4E is O‐GlcNAc‐modified at Threonine 168 and Threonine 177. A, Flag‐eIF4E was purified from HEK293T cells co‐expressing with OGT by immunoprecipitated for western blotting (left panel) and coomassie blue staining (right panel). The arrow indicated Flag‐tagged eIF4E. B, Flag‐eIF4E was analysed by mass spectrometry. Mass spectrum of the doubly charged peptide IAIWT (GlcNAc T) ECENREAV (GlcNAc T) HIGRVYK showed O‐GlcNAcylation at Thr168 and Thr177. The b and y type product ions were marked on the spectrum. C, The level of eIF4E protein in Huh7 cells transfected with plasmids expressing wild‐type eIF4E or its O‐GlcNAcylation site mutant were analysed by western blotting. β‐actin expression was served as a loading control. D, The levels of exogenous eIF4E mRNA in Huh7 cells transfected with plasmids expressing wild‐type eIF4E or its O‐GlcNAcylation site mutant were analysed by quantitative reverse transcription PCR and normalized against β‐actin. Error bars represent *±*SD of triplicate experiments. The two‐tailed Student's *t* test was used. n.s, no significance. E, Huh7 cells were transfected with plasmids expressing wild‐type eIF4E or its O‐GlcNAcylation site mutant before CHX (10 μg/mL) was added and treated for indicated durations. Levels of exogenous eIF4E were determined by western blotting and normalized against β‐actin. The bottom panel showcases relative protein amounts of different groups. Error bars represent *±SD* of triplicate experiments. *P < 0.05. F, Huh7 cells transfected with plasmids expressing wild‐type eIF4E or its O‐GlcNAcylation site mutant were treated with MG132 (5 μg/mL) for 24 h. Exogenous eIF4E expression was examined using western blot analysis (bottom panel). β‐actin expression was served as a loading control. Exogenous eIF4E immunoprecipitated from Huh7 cells with anti‐FLAG M2 affinity gel were further examined with ubiquitination antibody (top panel)

To determine the location of the O‐GlcNAc site(s) on eIF4E by mass spectrometry analysis, Flag‐eIF4E was purified from HEK293T cells co‐expressing with OGT for western blotting and coomassie blue staining (Figure 4A). The eIF4E band was then in‐gel digested with both trypsin and chymotrypsin, and analysed by Nano‐LC‐ESI‐MS/MS MS analysis of purified eIF4E revealed that threonine 168 and threonine 177 in peptide IAIWTTECENREAVTHIGRVYK (amino acids163‐184) were O‐GlcNAcylated (Figure 4B). To further understand the function of eIF4E O‐GlcNAcylation at Thr168/Thr177, eIF4E O‐GlcNAcylation site mutants (T168A, T177A, T168,177A) were expressed in Huh7 cells. Mutation of eIF4E O‐GlcNAcylation site(s) at Thr 168 or/and Thr 177 down‐regulated eIF4E protein level without changing its mRNA level (Figure 4C‐D). O‐GlcNAcylation has been reported to participate in regulation of protein stability.^32^, ^33^ To further test the possibility that O‐GlcNAcylation protects eIF4E protein degradation via O‐GlcNAcylation site(s) at Thr 168 or/and Thr 177, half‐life of wild type, T168A, T177A, and T168,177A eIF4E were determined by cycloheximide chase experiment (Figure 4E). Wild‐type eIF4E but not the mutants, was found to display a longer half‐life. In addition, we examined whether O‐GlcNAcylation regulated eIF4E degradation through proteasome pathway. Huh7 cells stably expressing wild‐type eIF4E or its O‐GlcNAcylation site mutants were treated with the proteasome inhibitor MG132. Down‐regulation of eIF4E protein level by O‐GlcNAcylation site mutation could be reversed by treatment with MG132 (Figure 4F, bottom panel). We further detected the contribution of eIF4E O‐GlcNAcylation in its ubiquitination. Mutation of O‐GlcNAc‐modified eIF4E at Thr 168 or/and Thr 177 increased eIF4E ubiquitination (Figure 4F, top panel). Together, eIF4E O‐GlcNAcylation at T168/T177 protects eIF4E from degradation, resulting in increase of eIF4E protein level.

### 3.5 Knockdown of OGT reduces eIF4E expression and high expression of eIF4E predicts poor prognosis of HCC

**Figure 5:**
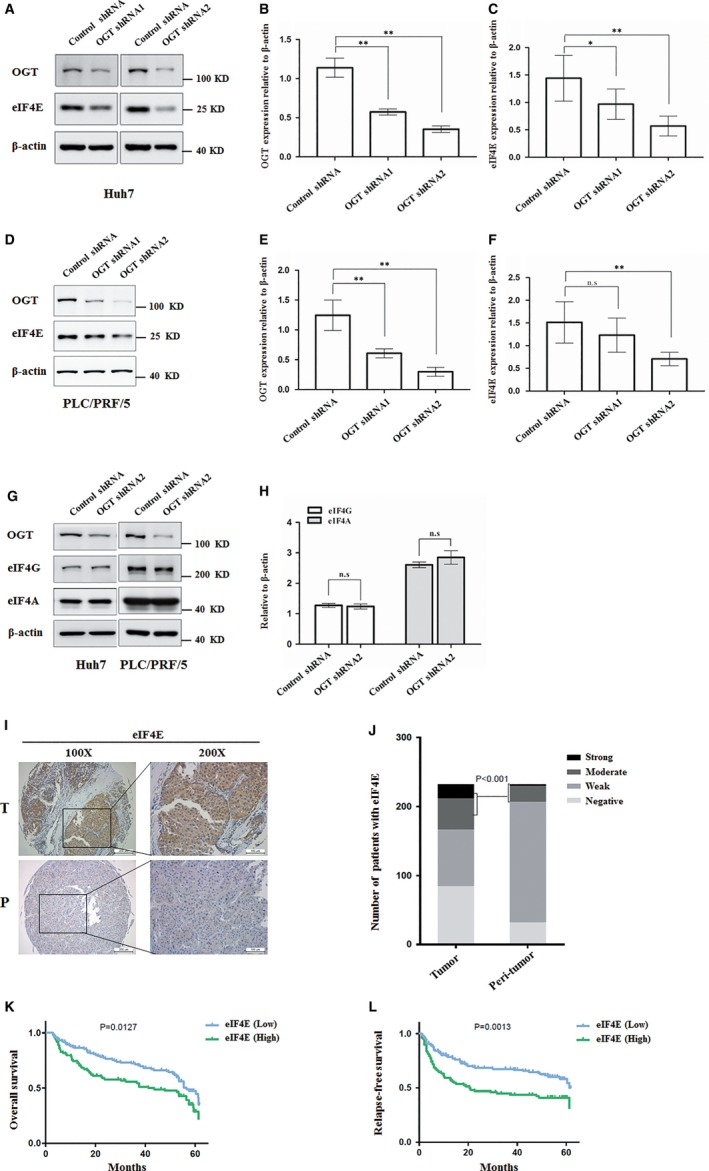
OGT knockdown reduces eIF4E protein expression and higher expression of eIF4E indicates a poor prognosis in HCC patients. A, The protein level of eIF4E was analysed by western blotting in Huh7 infected with control shRNA, OGT shRNA1 and OGT shRNA2 lentivirus. β‐actin expression was served as a loading control. B, OGT values were calculated from images in A. Data represent mean *± *SD of at least three independent experiments. **P < 0.01. C, eIF4E values were calculated from images in A. Data represent mean *± *SD of at least three independent experiments. *P < 0.05; **P < 0.01. D, The protein level of eIF4E was analysed by western blotting in PLC/PRF/5 cells infected with control shRNA, OGT shRNA1, and OGT shRNA2 lentivirus. β‐actin expression was served as a loading control. E, OGT values were calculated from images in D. Data represent mean *± *SD of at least three independent experiments. **P < 0.01. F, eIF4E values were calculated from images in D. Data represent mean *± *SD of at least three independent experiments. **P < 0.01; n.s, no significance. G, The protein level of eIF4A and eIF4G were analysed by western blotting in Huh7 and PLC/PRF/5 cells infected with control shRNA or OGT shRNA2 lentivirus. β‐actin expression was served as a loading control. H, eIF4G, and eIF4A values were calculated from images in G. Data represent mean *± *SD of at least three independent experiments. n.s, no significance. I, Representative immunohistochemical staining of eIF4E in liver cancer tissues and peri‐tumour liver tissues. J, Quantitative analysis of liver cancer tissue microarrays showed that the expression of eIF4E was higher in liver cancer tissues than in normal liver tissues (*P < *0.001). K, Kaplan‐Meier overall survival (OS) curve of HCC patients in correlation with expression of eIF4E. L, Kaplan‐Meier disease‐free survival (DFS) curve of HCC patients in correlation with expression of eIF4E. The DFS and OS rate significantly decreased in high expression of eIF4E (green line) compared to low expression of eIF4E (blue line)

The finding that O‐GlcNAcylation participated in eIF4E stability motivated us to investigate whether OGT regulated eIF4E protein level. OGT knockdown obviously reduced the level of eIF4E protein in Huh7 and PLC/PRL/5 cells (Figure 5A‐F). However, OGT knockdown did not significantly reduce the protein levels of eIF4A and eIF4G in translation initiation complex (Figure 5G‐H). Increasing evidences showed that eIF4E expression and activity were frequently elevated in various solid tumour types.^34^, ^35^, ^36^ Thus, we next performed immunohistochemistry assay to examine eIF4E expression in hepatoma. We first examined eIF4E expression by immunostaining in 232 HCC patients’ specimens. eIF4E expression level was markedly enhanced in tumour tissues compared to that in the peri‐tumour tissues (Figure 5I‐J). To further confirm the correlation of eIF4E with HCC prognosis, we compared overall survival (OS) and disease‐free survival (DFS) times between these two groups, as follows: patients with negative, weak, and moderate staining were stratified as the eIF4E low expression group, and those with strong staining as the eIF4E high expression group. Kaplan–Meier survival analysis showed that patients in the eIF4E high expression group had worse OS and shorter DFS than the eIF4E low expression group (Figure 5K‐L). Interestingly, multivariate analysis revealed that eIF4E intensity in tumours was an independent prognosticator for relapse‐free survival (RFS) and was significantly associated with clinic ‐pathologic features (serum alpha‐fetoprotein level, gamma glutamyl transferase, vascular invasion of HCC) (Table 1). Thus, OGT promotes eIF4E expression, and eIF4E expression is a valuable predictor for recurrence and survival in patients with HCC.

**Table 1:** Univariate and multivariate analysis of factors associated with survival and recurrence

Variables	Overall survival			Relapse‐free survival		
	Univariate *P *value	Multivariate		Univariate *P *value	Multivariate	
		HR (95% CI)	*p* value		HR (95% CI)	*p *value
Age, y (>52 vs ≤52)	0.479	NA		0.513	NA	
Gender (male vs female)	0.591	NA		0.671	NA	
HBsAg (positive vs negative)	0.600	NA		0.482	NA	
Liver cirrhosis (yes vs no)	0.920	NA		0.383	NA	
HCC family history (yes vs no)	0.101	NA		0.091	NA	
AFP, ng/mL (>20 vs ≤20)	0.004	1.55 (1.04‐2.30)	0.030	0.002	1.76 (1.14‐2.71)	0.010
ALT, U/L (>75 vs ≤75)	0.169	NA		0.050	NA	
γ‐GT, U/L (>54 vs ≤54)	<0.001	1.74 (1.17‐2.67)	0.006	<0.001	1.80 (1.15‐2.80)	0.009
Tumour size (>5 vs ≤5)	<0.001	1.96 (1.33‐2.91)	0.001	<0.001	1.91 (1.24‐2.94)	0.003
Tumour number (multiple vs single)	0.195	NA		0.133	NA	
Tumour capsule (yes vs no)	0.046	NS		0.055	NA	
Tumour differentiation (III‐IV vs I‐II)	0.004	NS		0.002	NS	
Vascular invasion (Yes vs No)	<0.001	1.86 (1.27‐2.72)	0.001	<0.001	1.71 (1.12‐2.61)	0.013
EIF4E (high vs low)	0.021	NS		0.002	1.67 (1.13‐2.45)	0.010

Univariate analysis was calculated by the Kaplan‐Meier method (log‐rank test). Multivariate analysis was done using the Cox multivariate proportional hazard regression model with stepwise manner (forward, likelihood ratio).

CI, confidential interval; HR, hazard ratio; NA, not adopted; NS, not significant.

### 3.6 OGT activates stem‐like cell potential of HCC cell through up‐regulation of eIF4E

**Figure 6:**
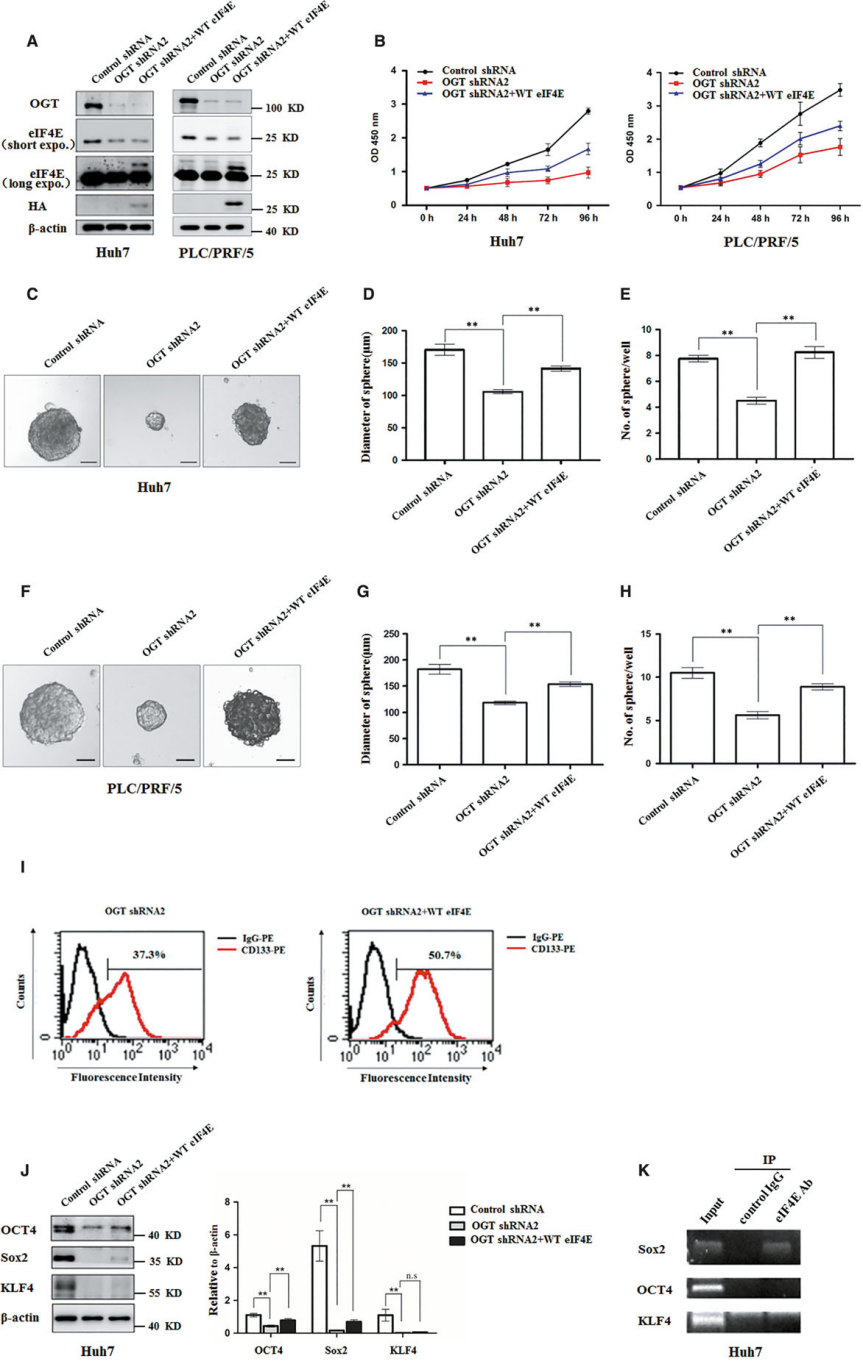
Knockdown OGT inhibits proliferation and tumorsphere formation of hepatoma cell through reducing eIF4E expression. A, Huh7 and PLC/PRF/5 cells were infected with control shRNA, OGT shRNA2 alone, or with wild‐type eIF4E lentivirus. The cell lysates were harvested for western blotting analysis using indicated antibodies. β‐actin expression was served as a loading control. B, Cell proliferation of Huh7 and PLC/PRF/5 cells infected with lentiviruses as in panel (A) were measured with CCK8 assay. (C‐H) Huh7 and PLC/PRF/5 cells infected with lentiviruses as in panel (A) were seeded into 96‐well plates. After 12 d, tumorsphere were counted and quantified. Representative images of sphere (scale bars, 100 μm) were shown (C, F). The diameter of sphere (D, G) and number of sphere (E, H) were count. Data represent mean *± *SD of at least three independent experiments. The two‐tailed Student's *t* tests were used. **P < 0.01. I, Huh7 cells expressing either OGT shRNA2 alone or with wild‐type eIF4E lentivirus were incubated with PE‐labelled anti‐AC133 antibody. The percentages of CD133^+^ cells in graphs were analysed by flow cytometry. Black line, control IgG staining; red line, CD133 staining. J, Cell lysates were examined by western blotting with indicated antibodies. The right panel showcases relative protein amounts of different groups. Error bars represent *±*SD of triplicate experiments. **P < 0.01; n.s, no significance. K, Huh7 cells were collected and subjected to immunoprecipitation with antibody against eIF4E or normal mouse IgG. Total RNAs were purified from immunocomplexes and subjected to RT‐PCR to measure Sox2, OCT4, and KLF4 mRNAs associated with eIF4E

Next, we examined whether OGT activated stem‐like cell potential of HCC cell through up‐regulation of eIF4E. To address this point, exogenous eIF4E was introduced into Huh7 and PLC/PRF/5 cells infected with OGT shRNA lentivirus (Figure 6A). CCK8 assay showed that the inhibitory effect of OGT knockdown on the proliferation of hepatoma cell was rescued by expression of exogenous eIF4E (Figure 6B). Furthermore, ectopic expression of eIF4E partly recused the inhibitory effect of OGT depletion on the diameter and number of tumorsphere formation (Figure 6C‐H). Flow cytometry analysis showed that the percentage of CD133^+^ cells increased from 37.3% to 50.7% after overexpression of eIF4E in Huh7 cells (Figure 6I). Consistent with this, ectopic expression of eIF4E in the OGT knockdown Huh7 cells increased the expression of stem‐like cell potential proteins (Sox2, OCT4) (Figure 6J). Considering that eIF4E activates gene translation through binding to the corresponding 5'‐untranslated region (5'‐UTR) sequences,^31^ we next addressed whether eIF4E bound to the 5'‐untranslated region of stem‐like cell potential proteins (Sox2, OCT4, and KLF4). RNA‐ChIP assay showed that eIF4E tightly bound to 5ʹ‐UTR of Sox2 mRNA (Figure 6K). Collectively, our data demonstrate that O‐GlcNAc modification was involved in the regulation of stem‐like cell potential through modification of eIF4E by O‐GlcNAc.

### 3.7 Glucose promotes cell proliferation and stem‐like cell potential of hepatoma cell through OGT

**Figure 7:**
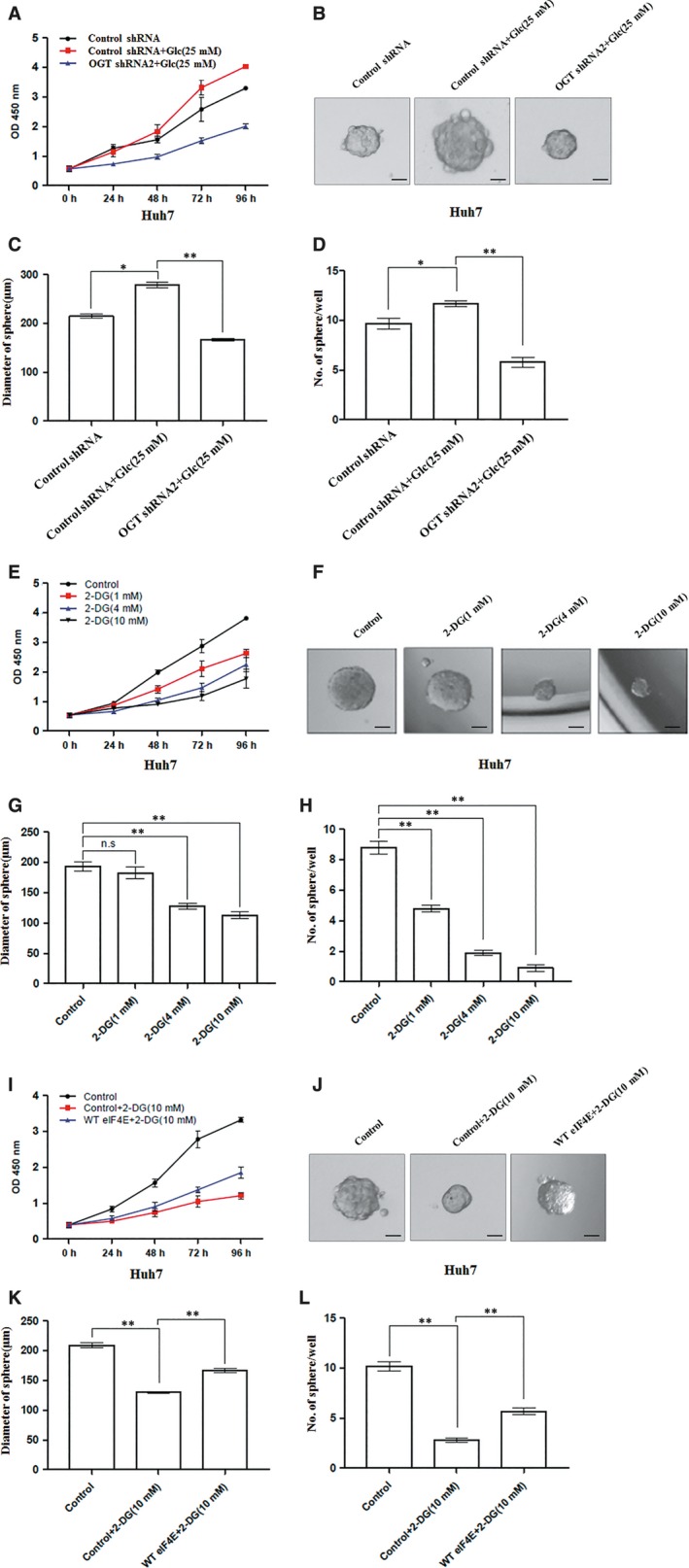
Glucose regulates cell proliferation and tumorsphere formation of hepatoma cell through OGT. A, Cell proliferation of Huh7 cells infected with control shRNA, OGT shRNA2 lentivirus and then treated with or without glucose (25 mM) were measured with CCK8 assay. (B‐D) Huh7 cells as in panel (A) in conditional culture treated with or without glucose (25 mM) were seeded into 96‐well plates. After 12 d, tumorsphere were counted and quantified. Representative images of sphere (scale bars, 100 μm) were shown (B). The diameter of sphere (C) and number of sphere (D) were count. (E) Cell proliferation of Huh7 cells treated with different concentrations of 2‐DG were measured with CCK8 assay. (F‐H) Huh7 cells treated with different concentrations of 2‐DG were seeded into 96‐well plates. After 12 d, tumorsphere were counted and quantified. Representative images of sphere (scale bars, 100 μm) were shown (F). The diameter of sphere (G) and number of sphere (H) were count. I, Cell proliferation of Huh7 cells infected with control, WT eIF4E lentivirus and then treated with or without 2‐DG (10 mM) were measured with CCK8 assay. (J‐L) Huh7 cells as in panel (I) were seeded into 96‐well plates in conditional culture treated with or without 2‐DG (10 mM). After 12 d, tumorsphere were counted and quantified. Representative images of sphere (scale bars, 100 μm) were shown (J). The diameter of sphere (K) and the number of sphere (L) were count. Data are quantified and presented as three independent experiments. The two‐tailed Student's *t* tests were used. *P < 0.05; **P < 0.01; n.s, no significance

High glucose increases intracellular concentrations of UDP‐GlcNAc, resulting in increased global O‐GlcNAcylation.^7^ We examined whether glucose promoted cell proliferation and stem‐like cell potential of hepatoma cell through OGT‐eIF4E axis. High glucose promoted cell proliferation and tumorsphere formation of Huh7 cells. However, OGT knockdown significantly reduced the positive effect of high glucose on cell proliferation and tumorsphere formation of Huh7 cells (Figure 7A‐D). Next, we determined the importance of eIF4E in glucose regulating stem‐like cell potential of hepatoma cell. 2‐Deoxy‐D‐glucose (2‐DG), a glucose analog, targets glucose metabolism and has been tested in multiple studies for possible application as an anti‐cancer therapeutic agent.^37^ We examined whether cell proliferation and stem‐like cell potential of hepatoma cell were inhibited by 2‐DG in Huh7 cells. We found that cell proliferation and stem‐like cell potential of Huh7 cells were obviously reduced with 2‐DG treatment (Figure 7E‐H). Ectopic expression of eIF4E rescued cell proliferation and tumorsphere formation of Huh7 cells treated with 2‐DG (Figure 7I‐L). These results indicate that OGT‐eIF4E axis contributes to activation of stem‐like cell potential of hepatoma cell by high glucose.

## 4 DISCUSSION

We aimed to elucidate the contribution and mechanism of O‐GlcNAcylation in hepatoma development. First, OGT knockdown attenuated not only the ability of proliferation but also stem‐like cell potential of hepatoma cell. Second, OGT modified the translation key regulator eIF4E with O‐GlcNAc at T168 and T177, protecting it against proteasomal degradation and increasing eIF4E protein stability. Third, the reduction in stem‐like cell potential effectors by down‐regulation of OGT was partially restored by eIF4E overexpression. Together, OGT promotes hepatoma cell proliferation and stem‐like cell potential at least partly through stabilization of eIF4E expression.

An interesting finding is that O‐GlcNAcylation regulates the stem‐like cell potential of Huh7 and PLC/PRF/5 cells. Abundant reports have showed that elevated O‐GlcNAcylation occurs in human malignancy and promotes tumour growth.^16^, ^17^ Consistent with this, OGT knockdown attenuated the ability of proliferation in hepatoma cell. Interestingly, down‐regulation of OGT expression inhibited the tumorsphere formation of hepatoma cell. Furthermore, down‐regulation of OGT expression reduced the expression of stem‐like cell potential proteins (Sox2, OCT4 and KLF4). Recent studies demonstrate that blocking O‐GlcNAcylation disrupts ESC self‐renewal. Upon embryonic stem cell differentiation, O‐GlcNAcylation on OCT4 at T228 is important to maintain embryonic stem cell self‐renewal.^38^ Our data showed that OGT activated stem‐like cell potential in hepatocarcinoma. To our knowledge, this is the first report that O‐GlcNAcylation contributes to stem‐like cell potential of hepatoma cell. However, the difference of O‐GlcNAcylation in normal stem cell and cancer stem cell should be further investigated.

OGT activated stem‐like cell potential in hepatoma cell partly through up‐regulation of eIF4E expression. The eukaryotic translation initiation factor 4E is a key regulator of protein synthesis, which is generally the rate‐limiting factor recruits mRNAs to eIF4F.^30^ Uncontrolled of eIF4E activity or expression in various cancers stimulates cellular proliferation and malignant transformation.^39^, ^40^ Thus, eIF4E has been considered as a therapeutic target in cancer. Previous studies indicate that eIF4E regulates function of common tumour cells.^40^ Here, we found that ectopic expression of eIF4E increased the diameter and number of tumorsphere and increased the expression of stem‐like cell potential proteins (Sox2, OCT4). Furthermore, 5ʹ‐UTR of Sox2 mRNA but not OCT4 mRNA, was tightly bound to eIF4E by RNA‐ChIP assay. The literature suggest that cellular mRNAs most sensitive to alterations in eIF4E availability and eIF4F complex formation. In tumours, elevated eIF4E function selectively and disproportionately increases translation of weak mRNAs. These mRNAs with G/C‐rich 5ʹ‐UTR had encoded potent growth, and survival factors notoriously involved in malignancy.^40^ Accordingly, we found that 5ʹ‐UTR of Sox2 had rich G/C bases compared to 5ʹ‐UTR of OCT4. Our data indicate that eIF4E regulates the stem‐like cell potential of hepatoma cell, providing a new mechanism that eIF4E promotes cancer development.

Our data also provide evidence that O‐GlcNAcylation increases the stability of eIF4E protein. The activity or expression of eIF4E is controlled by its binding proteins and by upstream signalling pathways. For example, phosphorylation of eIF4E on S209 by MNK1/2, released eIF4E from eIF4E‐binding proteins (4EBP1), resulting in the activation of eIF4E.^41^ Phosphorylation of eIF4E on S209 is elevated in human cancer and is associated with tumour aggressiveness and poor patient outcome.^42^ In addition, eIF4E is ubiquitinated at Lys159, suggesting the proteasome‐dependent proteolysis of eIF4E.^43^ Increasing researches suggest OGT participates in protein stability.^32^, ^33^ In this study, O‐GlcNAcylation of eIF4E at Thr168 or Thr177 protected it against proteasomal degradation and increased eIF4E protein stability. However, mutation at Thr168 and Thr177 did not completely abolish eIF4E O‐GlcNAcylation, indicated that other O‐GlcNAc site(s) was not detected by MS Other O‐GlcNAcylation site(s) of eIF4E needs to be further identified. Even so, eIF4E O‐GlcNAcylation regulates its protein stability, proving a linking between high glucose and cell proliferation.

In summary, we have described a novel role for the metabolic sensor OGT in the growth and stem‐like cell potential of hepatoma cells. OGT activates stem‐like cell potential in hepatocarcinoma through O‐GlcNAcylation of eukaryotic initiation factor 4E. These results provide a mechanism of HCC development. Furthermore, our findings that OGT‐eIF4E axis contributes to high glucose activating hepatoma cell stem‐like cell potential might provide a cue of the pathogenesis of HCC with diabetes.

## CONFLICT OF INTEREST

The authors declare that they have no conflicts of interest with the contents of this article.

## AUTHORS’ CONTRIBUTIONS

CBJ, XY, LCJ, YF, LYN, YTX participated in the experiments. CBJ drafted the manuscript. CBJ and DM performed histological classification. JJH, WYY, and GQ designed the study. All authors contributed to manuscript editing and approval.
